# Haptoglobin and Its Related Protein, Zonulin—What Is Their Role in Spondyloarthropathy?

**DOI:** 10.3390/jcm10051131

**Published:** 2021-03-08

**Authors:** Magdalena Chmielińska, Marzena Olesińska, Katarzyna Romanowska-Próchnicka, Dariusz Szukiewicz

**Affiliations:** 1 Department of Biophysics and Human Physiology, Medical University of Warsaw, Chałubińskiego 5, 02-004 Warsaw, Poland; katarzyna.prochnicka@gmail.com (K.R.-P.); dszukiewicz@hotmail.com (D.S.); 2 Department of Connective Tissue Diseases, National Institute of Geriatrics, Rheumatology and Rehabilitation, Spartańska 1, 02-637 Warsaw, Poland; marzena.olesinska@vp.pl

**Keywords:** haptoglobin polymorphism, inflammation, pathogenesis, spondyloarthropathy, zonulin

## Abstract

Haptoglobin (Hp) is an acute phase protein which supports the immune response and protects tissues from free radicals. Its concentration correlates with disease activity in spondyloarthropathies (SpAs). The Hp polymorphism determines the functional differences between Hp1 and Hp2 protein products. The role of the Hp polymorphism has been demonstrated in many diseases. In particular, the Hp 2-2 phenotype has been associated with the unfavorable course of some inflammatory and autoimmune disorders. Its potential role in modulating the immune system in SpA is still unknown. This article contains pathophysiological considerations on the potential relationship between Hp, its polymorphism and SpA.

## 1. Introduction

Spondyloarthropathy is one of the most common rheumatic diseases whose prevalence varies between 0.4 and 1.9% in different countries [1]. The heterogeneity of SpA is the result of numerous overlapping environmental and genetic factors which make the overall pathogenesis of the disease still elusive.

The dominant role of the innate immune system in the pathophysiology of SpA indicates its autoinflammatory character rather than an autoimmune one, although it is ultimately classified as an immune-mediated disease [2]. Several cytokine pathways are involved in the inflammatory process in SpA but it is the increased production of free oxygen radicals that directly corresponds to the destruction of tissues. Increased oxidative stress and its link to disease activity was demonstrated in the prototype form of SpA-ankylosing spondylitis (AS) [3].

Hp is a molecule that regulates the immune response and reduces oxidative stress; thus, its role may be crucial in the inflammatory pathways implicated in SpA [4]. Increased local expression of Hp was described in arthritis [5]. Hp is described as an inhibitor of collagen degradation and an important factor in cell migration [6]. Both of these processes characterize arthritis.

Human Hp is α^2^-sialoglycoprotein that belongs to acute phase proteins. Its synthesis, mainly in hepatocytes, is stimulated by proinflammatory cytokines IL-1, IL-6 and tumor necrosis factor α (TNFα). There are three major Hp phenotypes: Hp 1-1, Hp 2-1 and Hp 2-2, which arise from diversity in α chain compositions. Hp phenotypes differ in molecular size and structure, which determine their biological properties. The main role of Hp is connected with the binding capacity of hemoglobin (Hb). It forms a soluble complex with Hb, which is not filtered in the kidneys but is broken down in the liver, thus preventing kidneys from damage. Moreover, Hp dampens the inflammation by inhibiting the synthesis of prostaglandins, leukotrienes and cathepsin B. It reduces oxidative stress of iron-derived reactive oxygen connected with free Hb release. Hp 2-2 is the least effective in terms of antioxidative and anti-inflammatory activities due to its polymeric form weakly moving to the tissues [4,7,8].

Elevated serum Hp concentrations and a positive correlation between Hp levels and disease activity parameters have been observed in SpAs. Hp is an inflammatory marker used in the evaluation of treatment efficacy in many clinical trials [9,10,11]. An interesting issue is how Hp shapes the anti-inflammatory response in SpA and whether there are significant differences between Hp phenotypes in this respect. It also seems important for Hp2 gene carriers as the Hp2 precursor (pre-Hp2) molecule, zonulin, was upregulated in a recently conducted study in AS patients [12].

This article will focus on pathophysiological mechanisms in SpA in which Hp and its polymorphism may be crucial. We propose that Hp and its related protein, zonulin, may have important functions in the pathogenesis of SpA. Uncovering what role Hp and its polymorphism play in SpA would be advantageous in the future.

## 2. Distribution of Haptoglobin Phenotypes in Spondyloarthropathies

The distribution of general Hp alleles presents the frequencies of 0.4 for Hp1 and 0.6 for Hp2 in Europe [4]. The most widespread theory of evolutionary and structural biology of Hp states that the gene Hp2 occurred approximately 2 million years ago in India and is evolutionarily younger than the Hp1 gene [13]. Its spread beyond the Asian continent indicates the existence of certain advantages over the Hp1 allele. The hypothesis says that the appearance and increasing prevalence of the Hp2 allele is related to parasitic infections, especially malaria [14]. Nevertheless, in light of recent studies, this theory seems to be controversial, because it is also likely that the Hp2 gene is much older than previously assumed [15].

The anti-inflammatory properties of Hp depend on the phenotype and are the weakest for the Hp 2-2 phenotype. Hp 2-2 is associated with a higher predisposition to autoimmune and inflammatory diseases and worse outcomes of many of them [14,16,17,18]. The lower concentrations of Hp in serum and tissues in patients with inflammatory bowel diseases and Hp 2-2 phenotype may be associated with higher concentrations of proinflammatory cytokines compared to other patients [19].

The first published study on the distribution of Hp phenotypes in patients with rheumatoid spondylitis dates back to 1962. [20]. No statistical significance was found between the study group (45 Caucasian males) and the healthy volunteers, but it was noted that the differences in the distribution of the Hp1-1 phenotype between these two groups were at the borderline of statistical significance. The authors suggested conducting the study on a larger group of patients.

No significant difference in Hp frequency in AS (48 Caucasian individuals) and no correlation between Hp phenotype and serum C-reactive protein (CRP) were found by Sitton and Dixon [21]. The authors observed disturbed proportions of Hp 2-1 and Hp 2-2 phenotypes compared to patients with rheumatoid arthritis in favor of Hp 2-1. The relatively small size study is its limitation, as listed by the authors. Nothing is known about other factors that could affect the CRP value either. No data are given on, e.g., medicines taken by patients.

Baeten et al. investigated the expression of CD 163 (a scavenger receptor for Hb–Hp complexes) in patients with SpAs (130 Belgian residents) and showed no difference in the distribution of Hp phenotypes compared to both: the normal distribution in the Belgian population and between the subgroups of SpA [22]. The Hp 1-1 phenotype was weakly correlated with some of the disease activity parameters (CRP and erythrocyte sedimentation rate (ESR)) but not with the Bath Ankylosing Spondylitis Disease Activity Index (BASDAI) or with the Bath Ankylosing Spondylitis Metrology Index (BASMI).

The cross-sectional character of the study is its limitation. It is worth noting that the examined patients with SpAs were on various treatments modifying the course of the disease and some of them were untreated. Therefore, conclusions on the relationship between disease activity and Hp polymorphism are difficult to draw.

Surprisingly, different results from above were published by Soliev et al. from the Medical Institute in Tashkent [23]. The authors observed the significantly more frequent occurrence of the Hp 2-2 phenotype in ankylosing spondylitis, Hp 1-1 in psoriatic arthritis and Hp 2-1 in reactive arthritis. A total of 100 patients with SpAs, residents of Uzbekistan, were examined. The control group in this study had a similar distribution of Hp phenotypes to the European population. In the context of this research, it is worth noting the finding of Ciccia et al. who demonstrated upregulation of zonulin in the intestines of AS patients [12]. Zonulin is a relatively newly discovered molecule, having been identified in 2000 by Fasano and colleagues [24]. In 2009, zonulin was identified as pre-Hp2, an uncleaved form of mature Hp2 [25]. Importantly, the function of zonulin as a modulator of intestinal barrier tightness has been described. Until this discovery, pre-Hp2 was thought to be inactive. There are two identified zonulin triggers so far: gliadin and bacteria [26]. Of additional interest are the results of the proteomic analysis in AS performed by Liu et al. [27]. This study showed significantly increased expression of Hp precursor (pre-Hp) in AS patients compared to the control group. Moreover, it was shown that pre-Hp epitopes bind with high affinity to an allele HLA B*2705—the subtype which is the SpA risk factor for Caucasians. The authors found that the acute anterior uveitis is connected with an allele, HLA A*0201, especially when it coexists with HLA B*2705. HLA A*0201 possess properties that are particularly easy to combine with pre-Hp, similar to HLA B*2705.

Taken together, there has been no clear evidence of abnormal distribution of Hp phenotypes among people with SpA and there is no good data of the relationship between the Hp polymorphism and disease activity in SpA. However, the distribution of the Hp genes themselves seems to be relevant in the context of the functions that their precursors perform, especially the pre-Hp2- zonulin molecule, already well described in the literature.

## 3. The Role of Zonulin and Haptoglobin in Chronic Gut Inflammation

The idea that the gut joint axis is an important pathophysiological component of SpA is growing. Interestingly, studies have shown that inflammation of the intestines is correlated with the disease activity. Subclinical intestinal inflammation can be found in up to 68% of patients with SpAs [28]. Microscopic gut inflammation in axial SpA was described as related to younger age, progressive disease, male sex and higher disease activity [29]. In another study, the degree of bone marrow oedema in sacroilliac joints of patients with axial SpAs was linked to gut inflammation and male sex [30]. Gut inflammation seems to be a predictor of SpA progression to AS [28]. Inflammatory changes in the gut appear to be driven by an altered microbiome which causes an increased response from the immune cells especially through IL-23 cytokine release [31,32,33]. The pathogenic responsiveness to bacteria antigens and perturbation in the gut microenvironment may be associated with HLA B27 function [34,35,36]. Impaired intestinal barrier which leads to increased intestinal permeability has been demonstrated in SpA [12,36,37]. It is likely that the translocation of bacterial products plays an important role in the initiation of inflammation in the joints and in the uvea [38,39,40]. Marquez et al. revealed a protective function of Hp in the intestines [19]. Hp deficient mice developed severe inflammatory colitis with a particularly high production of IL 17. In this study, a higher frequency of Hp2 gene in the group with inflammatory bowel diseases than in the control group was presented.

Fasano et al. showed that zonulin regulates the tight junctions in the intestines and that increased gut permeability is associated with zonulin expression [41]. Ciccia et al. had similar observations [12]. The authors studied gut vascular and epithelial barrier impairment in AS patients and found that downregulation of endothelial and epithelial tight junction proteins is associated with zonulin. It was shown that high serum levels of lipopolysaccharide, lipopolysaccharide (LPS)-binding protein and intestinal fatty acid-BP together with the zonulin modified the activity of peripheral blood monocytes. This study demonstrated that zonulin, due to its affinity for the CD 163 receptor, leads to expansion of CD 163+ c-MAF + monocytes compatible with M2 macrophages. M2 macrophages were shown to be expanded in the peripheral blood, the gut and the synovium in patients with SpAs [42]. In another study, the number of macrophages CD 163+ (M2) in the synovium of patients with SpAs correlated with the disease activity and decreased during anti-TNF therapy [43].

The role of the microbiome in the development of SpA was shown in a study on transgenic HLA B27 rats [44]. The lack of commensal bacteria effectively protected them from the development of arthritis. Ciccia et al. demonstrated that ileal inflammation and perturbation in epithelial tight junctions in HLA B27 positive rats could be reversed by antibiotic treatments [12]. In another study, mice deprived of the normal Toll-like receptor 4(TLR4)—the lipopolysaccharide (LPS) sensor—were less likely to develop arthritis [45]. Additionally, the levels of proinflammatory factors in their synovial tissue were lower. TLR 4 was necessary to induce LPS-dependent arthritis.

LPSs increase the expression of the IL-23p19 gene in dendritic cells as well as activate innate lymphoid cells type 3 (ILC3) [46]. Polarization of innate lymphoid cells towards ILC3 may also result from the direct action of IL-23 [47]. Increased amounts of ILC3 were detected in the inflamed gut of patients with AS and correlated with disease activity [48]. ILC3 expresses integrin α4β7 and thus provides circulation between the intestine and the active inflammatory sites such as bone marrow and joints rich in α4β7 ligand [33,48].

On the other hand, innate immune cells, such as macrophages, NK cells, and neutrophils that are involved in intestinal inflammation, have receptors for Hp-Mac-1 leucocyte integrin b2 (CD11b/CD18) [49]. CD11b/18 together with other receptors is involved in regulating gene expression in response to LPSs. Further, Ling Zeng showed that macrophages in AS patients produce more IL-23 and TNF α in response to LPSs than in the control group. For some reason, these cells are particularly easily activated by LPS [50]. Hp was shown to dampen the LPS driven immune response mainly by inhibiting the monocyte and macrophage functions. This effect was selective and was associated with a decrease in production of TNF α, IL-10 and IL-12p70 [51].

An interesting issue in the context of differences in the course of SpA between men and women is the gender-specific anti-inflammatory properties of Hp in response to bacterial LPS, as was shown in the in vitro study. Raju et al. investigated changes in Hp levels in relation to the presence of LPS, TNF alpha and sex hormones [52]. The study showed that Hp was responsible for the endotoxin tolerance (ET) state, caused by a fall in TNF α levels, and was reversible when Hp was blocked. Hp suppressed the proinflammatory cytokines, released in response to bacterial LPSs, more strongly in the presence of estrogen. The opposite effect was observed by adding testosterone to the test blood, which caused an increase in TNF α. The authors conclude that this finding is consistent with observations of worse prognoses in the case of bacterial sepsis in males. On the other hand, higher levels of estrogen in females have long been suggested to correlate with a higher incidence of autoimmune diseases. The question is, can this be related to more frequent occurrence of AS among men and faster progression of the disease in their case?

To summarize, microbiomes, increased intestinal permeability and inflammation play important roles in the pathogenesis of SpA, as indicated by numerous reports from the literature. The regulation of response to LPS involves Hp and zonulin. There are some differences in the pattern of this reaction between the Hp phenotypes and it seems that the response is gender-specific. Zonulin in AS patients was shown to be linked with an impaired gut barrier, which may indicate a worse course of the disease in carriers of the Hp2 gene.

## 4. The Role of Haptoglobin in Inflammatory Pathways

The IL23/IL17 axis is an important cytokine pathway in SpA and a crucial part of antibacterial immunity [53]. One of the main sources of IL-23 in SpAs are macrophages with receptor CD 163 (M2) [50]. Ciccia et al. showed that polarization of macrophages towards M2 may happen upon exposure to zonulin; thus, it may enhance the IL23/IL17 axis [12]. Prostaglandins create the next most important inflammatory pathway in SpA, which affects this axis. A number of studies have reported that prostaglandin E2 (PGE2) leads to an increase in IL-23 production by bone marrow-derived dendritic cells and an increase in IL-23 receptor expression on Th17 cells [46,54,55]. Therefore, PGE2 shifts the immune response towards Th17. Additionally, IL-23 was shown to stimulate Th17 cells to produce PGE2 [56]. The recent studies have demonstrated that overexpression of prostaglandin E2 receptor 4 (EP4) on Th17 lymphocytes and monocytes is associated with disease activity and progression in AS [57,58]. Moreover, prostaglandins play an important role in new bone formation by EP2 and EP4 receptors [59]. Cyclooxygenase (COX) inhibition by nonsteroidal anti-inflammatory drugs has proven to be effective in SpA treatment [60,61].

Shim has shown that prostaglandins stimulate Hp synthesis [62]. On the other hand, Hp blocks COX. This process is phenotype-dependent and is the least pronounced for Hp 2-2 [4]. However, there are no data showing that inhibition of prostaglandin synthesis by Hp and that phenotype strength of this process have any clinical implications in SpA. This is a particularly interesting issue in terms of new bone formation.

Hp itself was demonstrated to modulate the response of lymphocytes, Th 17 [19]. It is unknown whether this process is phenotype-dependent. Moreover, Arredouani et al. showed a direct effect of Hp on T lymphocytes by significantly stronger suppression of Th2 cytokines (IL-4, IL-5, IL-10, IL-13) than Th1 cytokines (IFN gamma and IL-2) [63]. This outcome was observed for both Hp 1-1 and Hp 2-1 phenotypes. Unfortunately, the Hp 2-2 phenotype has not been studied for this purpose.

To conclude, Hp interacts with the receptors of immune cells and clearly takes part in the cytokine pathways important for SpA pathogenesis. A particularly interesting issue is the Hp phenotypic relationship of the prostaglandin synthesis blockade.

## 5. Haptoglobin and Oxidative Stress

Oxidative stress is based on increased production of reactive oxygen species (ROS) and the insufficiency of the system’s antioxidant potential to balance them. Inflammation can easily cause oxidative stress, but, on the other hand, ROS activate the genes involved in inflammation [64]. Thus, these two processes constitute a rather inseparable pathophysiological aspect.

One of the main sources of ROS are inflammatory cells. This way of defense against pathogens is particularly important for innate immune cells. Neutrophils and macrophage activation may lead to respiratory burst. Excessive generation of ROS may lead to cellular damage and death [65]. Interestingly, a study conducted on macrophages obtained from HLA B27-transgenic rats showed that stimulation of macrophages through IFN gamma and LPS leads to an increase in the production of ROS [66]. ROS reduction with antioxidant N-acetylcysteine significantly reduces transcriptions of proinflammatory cytokines.

In general, increased numbers of oxidative stress biomarkers were observed in patients with AS and PsA [67]. There are numerous reports from the literature showing that oxidative stress has an important function in AS pathogenesis [3,68]. There are hypotheses stating that high ROS toxicity may be responsible for articular cartilage damage and bone loss in AS [69]. Pathological bone formation may also be associated with Wnt/Beta-catenin and BMP/Smad pathways activation triggered by ROS [70]. The last meta-analysis of 2020 showed that some oxidative stress markers correlate with disease activity in AS [71]. In one study, it was observed that oxidative stress can be reduced by infliximab therapy [72].

The antioxidant properties of Hp are mainly related to its ability to form complexes with Hb. The smallest antioxidative capacity has the phenotype Hp 2-2 [4]. However, it seems that Hp is also a strong antioxidant, regardless of its ability to bind Hb. Additionally, Hp increases the resistance of the cell to oxidative stress and this property also seems phenotypically dependent [73]. Additionally, Hp can directly bind to neutrophils, inhibiting production of ROS and influencing their responses to other agonists [74]. It is interesting that TNF α, by stimulating p 55 receptors on neutrophils, leads to release of Hp [75]. Moreover, TNF α is one of the main factors responsible for the production of free oxygen radicals in AS [76].

Summarizing, Hp is produced during pro-oxidative conditions such as inflammation and has important antioxidant functions. Oxidative stress is another pathway in SpA, which may be influenced by Hp.

## 6. Conclusions and Research Directions

Despite the scientific basis on which Hp and its precursors are linked to immune response, there is little research on their influence in the pathogenesis of SpA. Considering that Hp and zonulin are associated with the immunological pathways distinctive for SpA, such studies could prove very worthwhile. It seems highly possible that interplay between genetic susceptibility to SpA and environmental factors may be prevented by intercellular tight junctions regulated by zonulin.

In this context, research using larazotide acetate seems interesting. Larazotide acetate (also known as AT-1001) is an oral synthetic peptide that blocks the action of zonulin by increasing the integrity of the intestinal barrier and reducing the immunoreactivity associated with its impairment [77]. Studies on AT 1001 in patients with coeliac disease showed reductions in gastrointestinal symptoms compared to those on a gluten-free diets alone [78] and during gluten challenge [79]. Would AT 1001 be effective in the therapy of SpA in Hp2 gene carriers?

**Figure 1:**
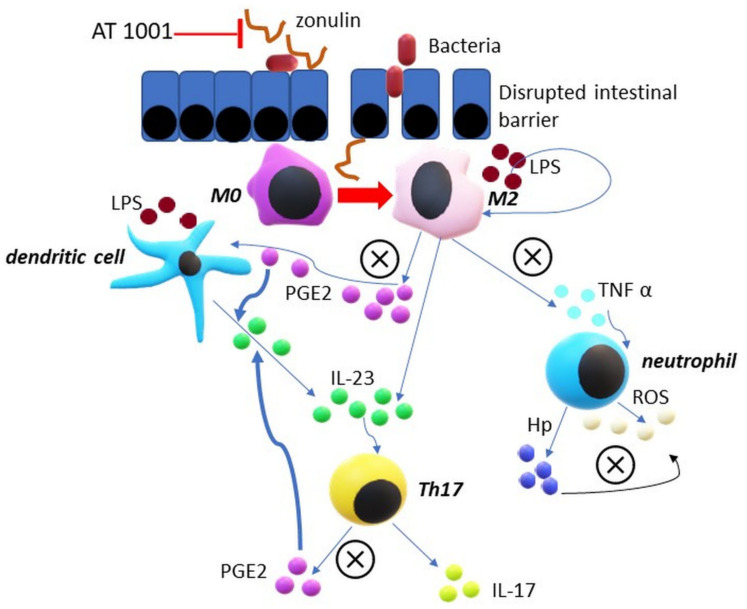
Proposed model of haptoglobin and zonulin impact on pathophysiology of spondyloarthropathies. Enteric bacteria stimulate zonulin secretion. Zonulin increases intestinal permeability and causes bacteria and lipopolysaccharides (LPSs) to penetrate the intestinal wall. LPSs lead to the polarization of macrophages towards M2, activate dendritic cells and boost production of proinflammatory cytokines by them. IL-23 strongly stimulates lymphocytes’ Th17 to secrete cytokine IL-17 and PGE2. PGE2 in turn strengthens the response of Th17 cells to IL-23 and increases production of IL-23 by dendritic cells. TNF is a powerful oxidative stress trigger but on the other hand leads to the release of Hp from neutrophils. Hp has antioxidant properties and reduces macrophage M2 response to LPSs by decreasing TNF alpha secretion. Hp also inhibits the production of prostaglandins in other signal pathways between cells. AT 1001 inhibits zonulin functions and may represent a novel therapeutic option in SpA. ⓧ = sites of haptoglobin inhibitory action.

Figure 1 depicts the immunological pathways in SpA regarding the mechanism of actions of Hp and pre-Hp2. These are the sites where Hp polymorphism may be relevant.

Returning to the topic of our work—haptoglobin and its related protein, zonulin—what is their role in spondyloarthropathy?—currently, we are not able to answer this question unequivocally on the basis of the available literature. There is very little research on the subject. However, we want to draw attention to the issue of potential immunomodulatory functions of Hp and zonulin in SpA. Research on this subject, especially with regard to the polymorphisms of Hp and pre-Hp2, could help us understand the difficult pathogenesis of this disease and to develop better and more effective methods of treatment.

Whether the enhancement of the natural barrier by the use of a zonulin blocker could inhibit the development of SpA and alleviate the course of the disease is questionable, although it is worth answering this question one day.
